# When 19 is greater than 92

**DOI:** 10.7554/eLife.01514

**Published:** 2013-10-15

**Authors:** Lauren R Zeitels, Joshua T Mendell

**Affiliations:** 1 **Lauren R Zeitels** is in the Medical Scientist Training Program, Johns Hopkins University School of Medicine, Baltimore, United Stateslzeitel1@jhmi.edu; 2 **Joshua T Mendell** is at the Department of Molecular Biology, UT Southwestern, Dallas, United StatesJoshua.Mendell@UTSouthwestern.edu

**Keywords:** microRNAs, c-Myc, Eμ-myc lymphoma, apoptosis, p53, Mouse

## Abstract

The gene *miR-17-92* encodes six different microRNAs, with one of these acting as an internal brake that opposes the oncogenic activity of the others in some cancer contexts.

**Related research article** Olive V, Sabio E, Bennett MJ, DeJong CS, Biton A, McGann JC, Greaney SK, Sodir NM, Zhou AY, Balakrishnan A, Foth M, Luftig MA, Goga A, Speed TP, Xuan Z, Evan GI, Wan Y, Minella AC, He L. 2013. A component of the *miR-17-92* polycistronic oncomir promotes oncogene-dependent apoptosis. *eLife* **2**:e00822. doi: 10.7554/eLife.00822

**Image** B-cell lymphomas overexpressing the entire *miR-17-92* cluster (left) exhibited more apoptotic cells than those overexpressing the *miR-17-92* cluster without *miR-92a* (right).

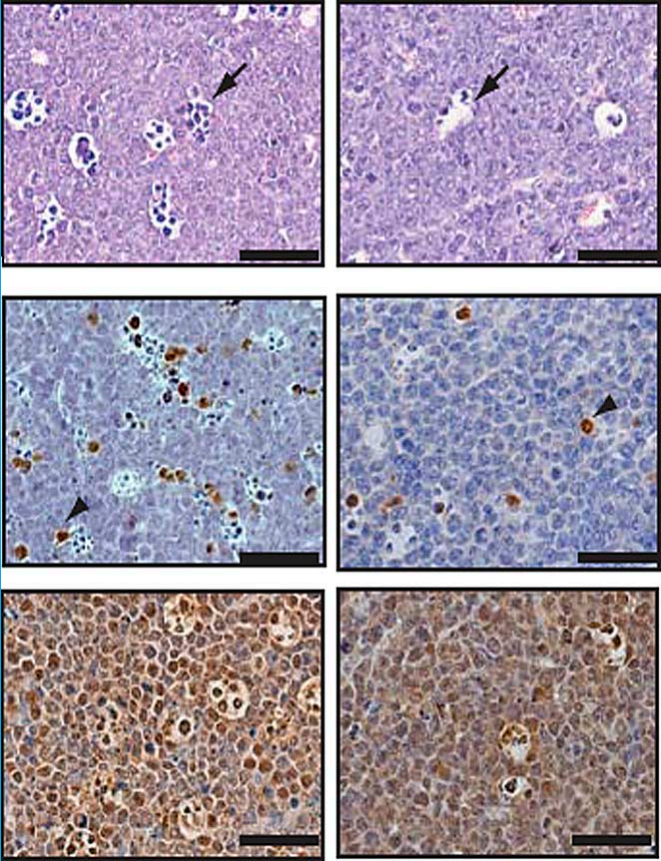

MicroRNAs are short, single-stranded RNA molecules that silence genes by targeting messenger RNA molecules. Examples of abnormal microRNA expression and function have been observed across the full spectrum of human cancers. These findings, coupled with data showing that microRNAs can promote or inhibit the formation of tumours in cell lines and animal models, have led to a widespread acceptance that some microRNAs function as *bona fide* oncogenes and tumour suppressors. In particular, the *miR-17-92* cluster—a group of six microRNAs all encoded by the same gene—has been studied extensively since researchers first discovered that it is overexpressed in Burkitt’s lymphoma, diffuse B-cell lymphoma and multiple types of solid tumours (Ota et al*.*, 2004).

The *miR-17-92* cluster is transcribed as a single primary transcript that is subsequently processed into six mature microRNAs: *miR-17*, *miR-18*, *miR-19a*, *miR-20*, *miR-19b*, and *miR-92a* (Figure 1). The assembly of multiple microRNAs into polycistronic transcripts has occurred repeatedly during evolution, but the functional significance of this type of organization has remained largely unexplored. Now, in *eLife*, Lin He of the University of California, Berkeley and colleagues—including Virginie Olive and Erich Sabio as joint first authors—provide new insights into the functional relationships between the individual components of the *miR-17-92* cluster (Olive et al., 2013).

**Figure 1.**
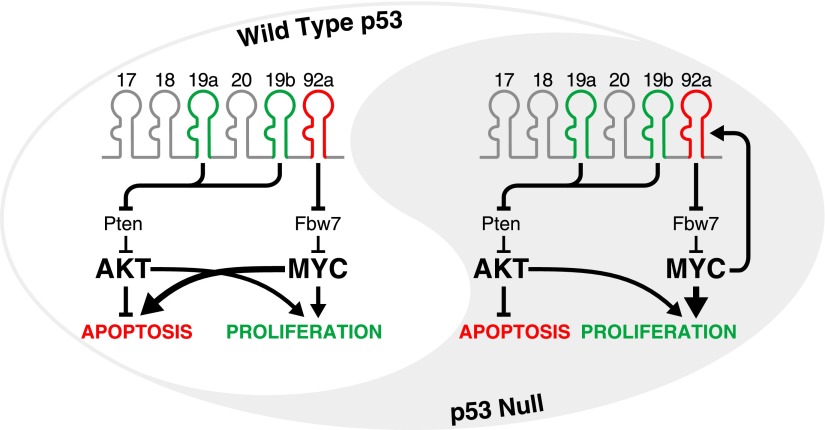
The yin and yang of *miR-92a* in tumours with and without an intact p53 pathway. The oncogenic activity of *miR-17-92* in the development of B-cell lymphomas is largely attributable to *miR-19*-mediated suppression of the tumour suppressor gene *Pten*, and the consequent activation of the PI3K/Akt pathway which inhibits apoptosis. At the same time, *miR-92a* causes hyperactivation of MYC by downregulating *Fbw7* (a gene that encodes an enzyme that facilitates MYC degradation). In cells with an intact p53 pathway (left), high levels of MYC promote proliferation but also engage the p53 pathway to trigger apoptosis, allowing *miR-92a* to counteract the pro-proliferative and pro-survival effects of *miR-19*. In the p53-deficient state (right), the hyperactivation of MYC does not lead to apoptosis. Instead, *miR-19* and *miR-92a* both promote the growth of tumours. Moreover, since MYC directly activates the transcription of *miR-17-92,* a feed-forward loop is established which may amplify these effects.

The oncogenic activity of *miR-17-92* was first directly demonstrated by He and co-workers using *Eμ-myc* mice (He et al*.*, 2005). MYC is a transcription factor that is involved in the regulation of a large number of genes and hyperactivation or amplification of the *Myc* gene occurs in many cancers. *Eμ-myc* transgenic mice overexpress the *Myc* gene in B lymphocytes, causing them to develop B-cell lymphomas by 4–6 months of age (Adams et al., 1985). He et al. showed that *miR-17-19b*—a truncated form of the cluster that does not contain *miR-92a*—accelerated the development of B-cell lymphoma when expressed in hematopoietic progenitor cells from *Eμ-myc* mice. Further experiments pinpointed *miR-19* (represented by *miR-19a* and *miR-19b* in the cluster) as the key oncogenic component of the cluster in this model (Olive et al*.*, 2009; Mu et al*.*, 2009) (Figure 1).

Olive, Sabio, He and co-workers have now further delineated the contributions of individual microRNAs within *miR-17-92* to the oncogenic activity of the cluster. Surprisingly, they found that *miR-92a* counteracts the effects of *miR-19* during the growth and development of B-cell lymphomas. This antagonistic function for *miR-92a* was first suggested by experiments which showed that the truncated cluster (which lacks *miR-92a*) exhibited significantly stronger oncogenic activity than the full cluster in *Eμ-myc* B cells. Similarly, mutations that inactivated *miR-92a* strongly enhanced the oncogenic effects of the full cluster. In pre-malignant B cells from *Eμ-myc* mice, and also in mouse embryonic fibroblasts, the expression of *miR-92a* triggered programmed cell death (apoptosis), thereby counterbalancing the effects of *miR-19* (which suppresses apoptosis). Intriguingly, however, the overexpression of *miR-92a* also increased the proliferation of fibroblasts and B cells. This dichotomy can be at least partially explained by the ability of *miR-92a* to reduce expression of the gene *Fbw7*, which encodes an enzyme that facilitates the degradation of the MYC protein. Thus, the increase in MYC levels resulting from *miR-92a* expression simultaneously stimulates proliferation and engages the p53 tumour suppressor pathway, resulting in apoptosis (Figure 1, left).

While the *miR-92a-Fbw7-*MYC axis clearly contributes to the apoptosis induced by *miR-92a*, it is unlikely that this mechanism can fully explain the ability of this microRNA to induce cell death. For example, elevation of MYC protein in fibroblasts does not cause apoptosis as efficiently as expressing *miR-92a*. Additionally, Olive et al. did not demonstrate that *Eμ-myc* lymphoma cells with enforced *miR-92a* expression have higher MYC levels than lymphoma cells without enforced *miR-92a*. Further studies will be necessary to establish precisely how this pathway influences the growth and development of B-cell lymphomas, and whether *miR-92a* leads to apoptosis via other pathways.

Olive et al. suggest that the ratio of *miR-19* to *miR-92a* determines the overall oncogenic potency of the *miR-17-92* cluster. Interestingly, they found that *miR-19* is more highly induced than *miR-92a* in pre-malignant and malignant *Eμ-myc* B cells compared to normal B cells. A similar phenomenon is evident in human Burkitt’s lymphoma cell lines. These observations suggest that the processing of *miR-19* and *miR-92a* from the *miR-17-92* primary transcript or the stability of the individual mature miRNAs is differentially regulated in normal B cells compared to lymphoma cells. Elucidation of this mysterious post-transcriptional regulatory pathway, which itself may be oncogenic, will undoubtedly be a priority for future work.

Given that the pro-apoptotic activity of *miR-92a* depends on the presence of an intact p53 pathway, it is important to consider the effects of expressing this microRNA when p53 is absent, as is the case in a large fraction of human tumours. In the absence of p53, the ability of *miR-92a* to enhance levels of the protein MYC will undoubtedly serve a pro-tumorigenic agenda. Indeed, *miR-92a* has been shown to promote the proliferation and survival of liver and colon cancer cell lines (Shigoka et al., 2010; Tsuchia et al., 2011). Moreover, given that MYC leads to increased transcription of *miR-17-92* (O’Donnell et al., 2005), it is conceivable that the stabilization of MYC by *miR-92a* may result in a feed-forward loop that leads to even greater activation of MYC. In such a setting, *miR-92a* may work with, rather than against, *miR-19*, with both of these microRNAs promoting the formation and growth of tumours through independent pathways (Figure 1, right).

This work also begins to address a broader question: why have microRNA clusters evolved? One might expect that co-transcribed microRNAs would generally work in concert and, indeed, there are many such examples. For instance, both microRNAs within the *miR-199-214* cluster work together to promote cardiac remodelling (El Azzouzi et al., 2013). Nevertheless, the work of Olive, Sabio and colleagues—who include co-workers from the UK, China and universities across the US—demonstrates that co-transcription does not necessitate cooperation. The extension of their work to other clusters and other in vivo models will elucidate whether this intricate balance is unique to the *miR-17-92* cluster or if it can be generalized to other microRNAs and other species.
